# GPT-4o for Automated Determination of Follow-up Examinations Based on Radiology Reports from Clinical Routine

**DOI:** 10.1038/s41598-026-40317-9

**Published:** 2026-04-16

**Authors:** Kenan Kaya, Lukas Müller, Thorsten Persigehl, David Zopfs, Christian Nelles, Thomas Dratsch, Andra-Iza Iuga, Jan Paul Janssen, Thomas Schömig, Jonathan Kottlors, Carsten Gietzen, David Maintz, Lenhard Pennig, Tobias Jorg, Nedim Beste, Robert Terzis

**Affiliations:** 1 Institute for Diagnostic and Interventional Radiology, Faculty of Medicine and University Hospital Cologne, University of Cologne, Cologne, Germany; 2 Department of Diagnostic and Interventional Radiology, University Medical Center of the Johannes Gutenberg-University, Mainz, Germany; 3 Institute for Diagnostic and Interventional Radiology, University Hospital Cologne, Kerpener Str. 62, 50937 Cologne, Köln, Germany

**Keywords:** Artificial intelligence, Large language models, Oncologic imaging, Magnetic resonance imaging, Computed tomography, Diseases, Gastroenterology, Health care, Medical research

## Abstract

Follow-up imaging recommendations vary across radiologists despite established guidelines. We evaluated whether a large language model (GPT-4o) can standardize follow-up timing and modality selection from routine radiology reports. In this retrospective, two-center study, 100 CT/MRI cases (25 each: head/neck, liver, lung, pancreas) were randomly sampled. GPT-4o and two human readers (R1, resident; R2, board-certified) generated follow-up recommendations from report text. Expert consensus, blinded to rater, assessed completeness, appropriateness of imaging modality, timing accuracy, and global follow up quality on a 5-point scale, with 5 being the highest score. Median global quality was 4 (2–5) for GPT-4o, 4 (1–5) for R1, and 4 (1–5) for R2; GPT-4o exceeded R1 (*p* < 0.01) and did not differ from 2 (*p* = 0.06). Relative treatment effect showed a reader effect (GPT4o 0.56 vs. R1 0.43 vs. R2 0.51; *p* < 0.01) and no center effect (0.50 vs. 0.50; *p* = 0.91). Correctness of follow-up timing was 96% (96/100) for GPT-4o, 75% (75/100) for R1, and 90% (90/100) for R2 (*p* < 0.001 for GPT-4o vs. R1). Completeness of follow-up was 92% (92/100) for GPT-4o, 91% (91/100) for R1, and 80% (80/100) for R2 (*p* = 0.014 for GPT-4o vs. Reader 2). No significant differences were observed regarding appropriateness of imaging modality. GPT-4o generated follow-up recommendations with overall quality comparable to an experienced radiologist and superior to a trainee, with high completeness and generally appropriate follow up timing and modality, supporting its role as decision support for standardized, guideline-aligned follow-up.

**Supplementary Information:**

The online version contains supplementary material available at 10.1038/s41598-026-40317-9.

## Introduction

Follow-up imaging decisions are a critical component of radiology practice, ensuring that findings are monitored appropriately and managed according to best practices. In clinical practice, radiologists are responsible for evaluating the need for further imaging based on initial findings, for instance by recommending an MRI for a newly identified hepatic lesion or a CT scan for a pulmonary nodule. Various professional guidelines exist to standardize such decisions; for instance, the American College of Radiology (ACR) Incidental Findings Committee provides algorithms for managing incidental liver lesions^1^, and the Fleischner Society publishes criteria for follow-up of pulmonary nodules detected on CT^2^. Adherence to these guidelines is intended to ensure consistent, evidence-based recommendations for timing and modality of follow-up imaging.

However, in practice there is considerable variation and inconsistency in follow-up recommendations. Even when presented with similar findings, radiologists can differ markedly in whether they advise additional imaging and on what timetable. One large analysis found nearly a sevenfold inter-radiologist difference in the likelihood of recommending follow-up examinations, after adjusting for patient and exam factors^3^. Compliance with established guidelines is also suboptimal, for example in a survey of 834 radiologists, only about 35–61% of management decisions for small lung nodules were consistent with Fleischner Society recommendations^4^. Such variability in practice leads to potential inefficiencies: some patients may undergo unnecessary imaging (e.g. incurring avoidable cost and radiation exposure), while others might not receive timely follow-up when it is warranted^5,6^. These disparities highlight a need for decision-support tools to promote more standardized follow-up recommendations. Indeed, prior work has called for interventions like feedback on individual radiologists’ recommendation rates and increased guideline awareness to reduce unwarranted variation^7^. Yet implementing such measures uniformly in busy clinical settings remains challenging.

Recent advances in artificial intelligence (AI) offer a promising avenue to address this challenge. Radiology workflows traditionally generate text-rich reports that encapsulate all key findings and impressions; thus, they are well suited for modern natural language processing techniques. Large language models (LLMs) have opened new possibilities for analysing clinical text and supporting medical decision-making^8^. One prominent example is GPT-4o (Generative Pre-trained Transformer 4omni, OpenAI), an autoregressive LLM known for its ability to comprehend complex textual input and generate contextually appropriate, knowledge-informed responses^9^. These capabilities make GPT-4 and similar models attractive for clinical applications were understanding nuanced text, like a radiology report, and drawing on medical knowledge are required. Notably, LLMs have recently garnered significant attention in radiology informatics research, with studies demonstrating their potential across various tasks^10^. In experimental settings, GPT-4 and its predecessors have already shown encouraging results relevant to radiological workflow^11–13^.

Building on the potential of LLMs to interpret and triage radiological data, applying GPT-4o to automate follow-up imaging recommendations represents a logical progression. By harnessing GPT-4o’s ability to understand radiology report language and align it with established guideline knowledge, such a system could help reduce variability in follow-up decisions and alleviate the radiologist’s workload. Beyond radiology, LLMs are increasingly being evaluated in medicine for clinical documentation, decision support, and knowledge retrieval, and may facilitate scalable curation of imaging biobanks^14,15^.

Accordingly, the aim of our study was to evaluate GPT-4o’s performance in selecting appropriate follow-up intervals at the examination schedule, compared to a radiology resident and a board-certified radiologist across multiple subspecialties, using real-world radiology reports.

## Methods

### Ethics

This study received ethical approval (file number 24-1001-retro), and informed consent was waived by the institutional review board (Ethics Committee of the Faculty of Medicine, University of Cologne) due to the retrospective design. All procedures performed were in accordance with the ethical regulations and relevant guidelines of the institutional research committee. No patient-identifying information was provided to the artificial intelligence.

### Data acquisition and processing

Search criteria for this study were as follows:

1. Radiology reports for CT and MRI, both in- and outpatient, focusing on oncologic imaging.
2. Included examinations were restricted to the following subspecialties: head and neck, liver, lung, and pancreas.
3. Patient age ≥ 18 years.

Two radiology residents not participating in the subsequent evaluation of follow-up examinations performed stratified random sampling. For each center, all CT/MRI radiology reports fulfilling the predefined search criteria (oncologic imaging, head and neck, liver, lung, or pancreas; inpatient or outpatient) between October 2023 and June 2024 were retrieved from the radiology information system. Within each subspecialty stratum, reports were assigned consecutive study identifiers and screened in computer-randomized order until 25 eligible adult cases per subspecialty were included. In total, 103 reports were screened; three were excluded because the patients were under 18 years of age, resulting in a final cohort of 100 cases (71 MRI and 29 CT examinations; center 1: *n* = 60 cases; center 2: *n* = 40 cases). This balanced sampling strategy was chosen to cover multiple guideline frameworks across heterogeneous oncologic entities and is not intended to reflect the prevalence distribution of routine clinical case mix.

All radiology reports were submitted through our in-house radiology information systems (center 1: Orbis-RIS version 08.42, Dedalus Healthcare System Group DACH, center 2: RADCentre version 2024.07, Mesalvo GmbH) in German language. Each report included the patient’s medical history and a clearly defined clinical question, as documented by the referring departments in the radiology request form, together with the complete radiology report and final impression. This information was subsequently provided in textual format to GPT-4o, accompanied by a standardized prompt instructing the model to propose an appropriate follow-up examination.

### GPT-4o

GPT-4o was accessed through OpenAI’s web interface platform, ChatGPT (https://chat.openai.com/), between June and July 2024. Thus, prompt engineering was realized with *gpt-4o-2024–05–13*. Each text dataset was copied individually into a separate chat for analysis. GPT-4o evaluated each dataset using engineered prompting with zero-shot inference. For each case, a single output was generated without regeneration or post-hoc selection among multiple responses.

### Human readers

To compare GPT-4o’s performance with human readers, the datasets were reviewed independently by a radiology residents with 5 years (T.D.; R1) and a board-certified radiologist with 8 years (C.N.; R2) of experience. The evaluations were conducted without a specific time limit.

### Prompting

The prompt for the request to GPT-4o and human readers stated as follows:

*“Based on the medical history*,* imaging findings*,* and diagnostic assessment*,* specify the exact timeframe and imaging modality recommended for follow-up evaluation*".

Radiology request forms from four patients, one from each subspecialty, were used by non-evaluating contributors (R.T., K.K.) to iteratively refine the prompt over 10 development cycles. Each case was processed using a zero-shot prompting approach, with the RRFs individually entered into the ChatGPT web interface for evaluation. To assess prompt consistency, each of the four RRFs was processed five times. These four cases were randomly selected to represent each subspecialty and were not included in the final test set of 100 cases.

### Reference standard

Two board-certified radiologists with 25 years (T.P.) and 11 years (D.Z.) of experience independently reviewed and subsequently performed a consensus assessment of all text-based CT and MRI follow-up recommendations generated by GPT-4o and by the human readers (R1 and R2). Both reviewers were blinded to the source of each recommendation (GPT-4o vs. radiologists). Each recommendation was evaluated according to four predefined categories (I–IV), using binary and ordinal rating formats as described below:

I) Completeness of Follow-up Recommendation.

The overall completeness of each recommendation was assessed by determining whether all relevant pathologies requiring radiological follow-up were adequately addressed. This was rated as a binary variable (*Yes/No*), indicating the presence or absence of completeness.

II) Appropriateness of imaging modality

The correctness of the imaging modality suggested for follow-up examination was likewise rated using a binary scale (*Yes/No*), based on current clinical standards and diagnostic appropriateness for the given pathology or multiple pathologies, if applicable. When guidelines allowed more than one follow-up imaging modality, all guideline-supported modalities were rated as correct.

III) Accuracy of follow-up timing.

Timing accuracy was assessed in a three-tiered binary format:

- IIIa) Timing exactly correct (*Yes/No*).
- IIIb) Time range partially correct, no potential harm for the patient (*Yes/No*).
- IIIc) Timing completely incorrect; potential negative consequences for the patient (*Yes/No*).

Only one of these subcategories could be assigned per case, ensuring mutually exclusive classification. Timing correctness was assessed using the respective clinical guideline as the reference standard. Recommendations fully consistent with the guideline-defined timeframe were classified as exactly correct (IIIa). If the proposed timing partially overlapped with the guideline window and expert review did not identify potential negative clinical consequences, it was classified as partial correct/no harm (IIIb). Recommendations outside the guideline-supported timeframe and judged to entail potential harm were classified as incorrect (IIIc).

IV) Overall quality assessment

A global quality rating of the follow-up recommendation was performed using a 5-point Likert scale, defined as follows:

1. [completely incorrect and harmful].
2. [relevant errors/omissions with potential harm for the patient].
3. [multiple less relevant errors/omissions, no potential harm for the patient].
4. [single irrelevant errors/omissions; all pathologies addressed; no potential harm].
5. [no errors/omissions; timing and modality exactly correct].

To ensure a robust reference standard and minimize bias, all follow-up recommendations generated by GPT-4o, the radiology resident (R1), and board-certified radiologist (R2) were independently reviewed by two senior board-certified radiologists who served as expert raters as mentioned above. Each recommendation was evaluated for adherence to international guidelines from major radiological and oncological societies. In particular the National Comprehensive Cancer Network (NCCN)^16^, the American College of Radiology (ACR)^1^, the Fleischner Society^2^, the American Journal of Neuroradiology (AJNR)^17^, the German AWMF guidelines (Association of Scientific Medical Societies)^18^, the Fukuoka international consensus guidelines^19^, and the American Cancer Society (ACS) recommendations were consulted for their respective up-to-date follow-up protocols^20^. These sources cover a broad range of pathologies (Table 1); accordingly, the most frequently represented diseases in our cohort included intraductal papillary mucinous neoplasm (IPMN) of the pancreas (*n* = 15), hepatocellular carcinoma (*n* = 10), pancreatic carcinoma (*n* = 6), breast carcinoma (*n* = 5), malignant melanoma (*n* = 4), bronchial carcinoma (*n* = 3), and cholangiocarcinoma (*n* = 2). For each case, the expert consensus determined the appropriate guideline-based imaging surveillance and verified concordance of the human readers’ follow-up recommendations. An overview of all pathologies and their corresponding guideline sources is provided in Table 1.

**Table 1:** Frequency of occurrence of the different pathologies and disease types throughout the dataset with the corresponding guideline referred for evaluation of follow-up recommendation.

Pathology/disease type	Guideline	Frequency of occurrence
Intraductal Papillary Mucinous Neoplasm Carcinoma of the floor of the mouth Hepatic carcinoma Cancer of unknown primary (CUP) Pancreatic carcinoma Oropharyngeal squamosa cell carcinoma Breast carcinoma Asbestosis Oesophageal carcinoma Malignant melanoma Neuroendocrine Tumour Bronchial carcinoma Ovarian carcinoma Cholangiocarcinoma Adrenocortical carcinoma Cholesteatoma Parotid gland carcinoma Endometrial carcinoma Cervix carcinoma Osteosarcoma Urothelial carcinoma Testicular carcinoma Langerhans cell histiocytosis (LCH) Schwannoma Mucoepidermoid carcinoma Laryngeal carcinoma	Fukuoka ACS ACR and NCCN NCCN NCCN ACS NCCN AWMF NCCN NCCN NCCN NCCN and Fleischner NCCN NCCN NCCN AJNR NCCN NCCN NCCN NCCN NCCN NCCN NCCN NCCN NCCN NCCN	*n* = 15 *n* = 13 *n* = 10 *n* = 7 *n* = 6 *n* = 6 *n* = 5 *n* = 5 *n* = 4 *n* = 4 *n* = 3 *n* = 3 *n* = 2 *n* = 2 *n* = 2 *n* = 2 *n* = 2 *n* = 1 *n* = 1 *n* = 1 *n* = 1 *n* = 1 *n* = 1 *n* = 1 *n* = 1 *n* = 1

### Statistical analysis

All analyses were conducted in R (RStudio Version 2022.12). The primary ordinal outcome (Quality) was analyzed using the nparLD package for non-parametric factorial repeated measures designs with factors Reader, Center, and Examination, blocking on subject ID. We report relative treatment effects (RTE; 0–1), where 0.50 indicates no stochastic dominance relative to the pooled distribution, values > 0.50 indicate higher (better) quality, and values < 0.50 indicate lower quality. Omnibus p-values are provided for main effects and interactions. For planned within-subgroup comparisons, we performed Friedman tests with paired Wilcoxon signed-rank post-hoc tests; multiplicity was controlled using the Benjamini–Hochberg false discovery rate. Dichotomous secondary outcomes (e.g., correct modality, pathologies identified) were compared across Readers using Pearson’s χ² tests when assumptions were met and Fisher’s exact tests otherwise, applying Benjamini–Hochberg adjustment across families of tests. The Quality outcome is summarized as median [min–max] with cell counts; categorical outcomes are reported as percentages. Two-sided α = 0.05 was considered statistically significant. Prior to analysis, examination labels were translated into English and centers anonymized (“Center 1/2”). As an exploratory check, distributional shape was inspected using histograms and the Shapiro–Wilk test for Quality. To assess the robustness of the statistical analyses with respect to sample size, a sensitivity power analysis was conducted for paired reader comparisons. The analysis was performed assuming a two-sided significance level of α = 0.05 using a paired t-test approximation, with effect sizes expressed both in standardized units and in Quality score units based on the standard deviation of paired differences.

## Results

### Baseline characteristics

Demographic data were collected and analysed for a total of 100 patients (*n* = 53 female, *n* = 47 male). The selected RRFs included both in- (*n* = 62) and outpatients (*n* = 38). The average age of the participants was 60,7 ± 14,6 years (range, 18–85 years).

### Overall evaluation of the follow-up recommendations

**Fig. 1:**
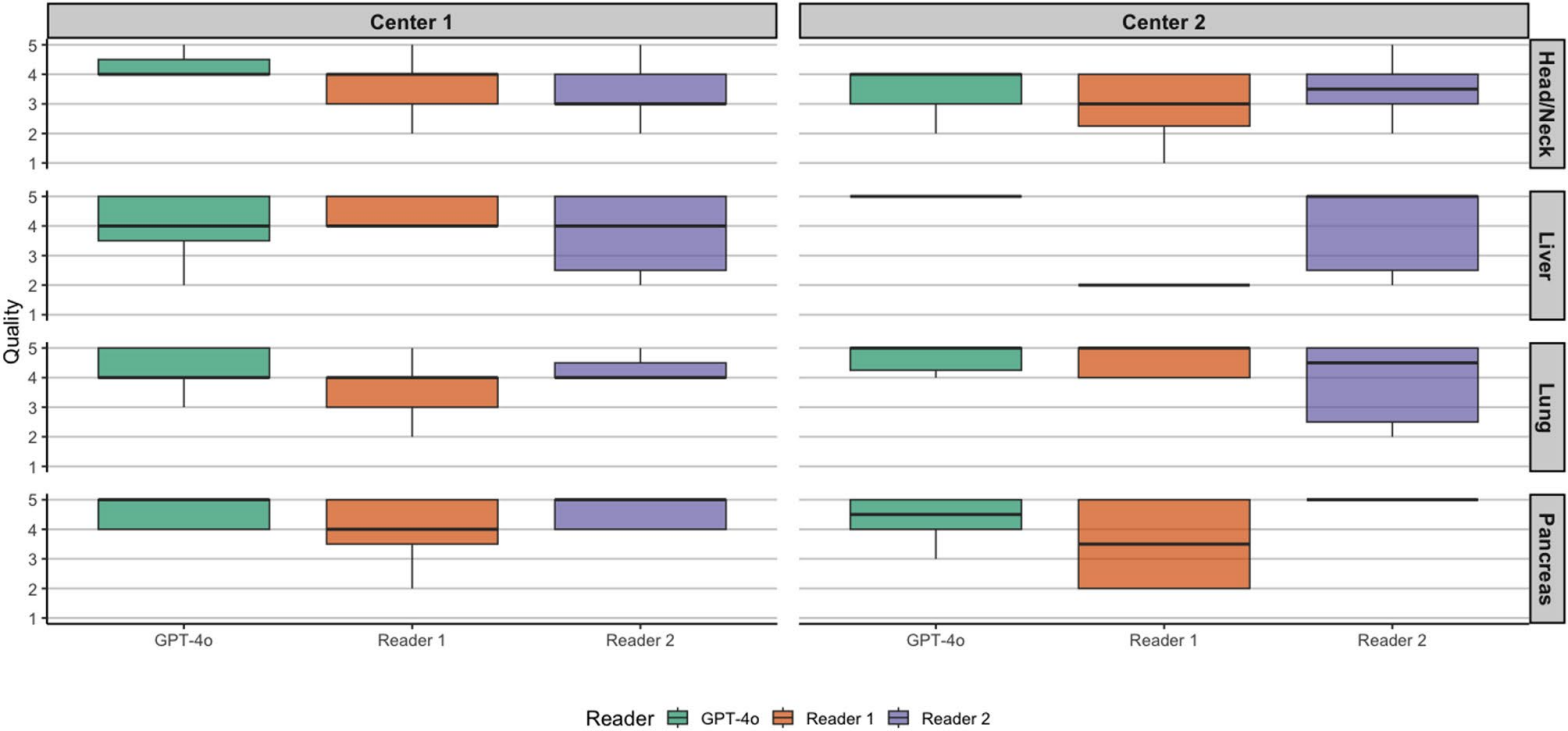
Visualization of overall follow-up quality by center. Reader 1 = Resident with five years of experience. Reader 2 = board-certified radiologist with eight years of experience.

Across 100 cases per rater, GPT-4o received a median global quality score of 4 (2–5), compared with 4 (1–5) for R1 and 4 (1–5) for R2; pairwise comparisons showed a significant higher quality distribution for GPT-4o versus R1 (*p* < 0.01), with no significant differences versus R2 (*p* = 0.06) or between R1 and R2 (*p* = 0.06) (Table 2/Fig. 1). In a non-parametric analysis of quality using relative treatment effects (RTE), the reader effect was significant (GPT-4o: 0.56; R1: 0.43; R2: 0.51; *p* < 0.01) (Table 3). A sensitivity power analysis for the paired reader comparisons indicated that, with the current sample size, 80% power at α = 0.05 was achieved only for effects at or above the reported minimum detectable effect. Accordingly, near-threshold results (e.g., *p* = 0.06) are compatible with a real but small effect. Full details of the sensitivity analysis are provided in the Supplementary Material.

**Table 2:** Summary of median values and ranges for the expert consensus quality ratings of the proposed follow up imaging recommendations; with corresponding p-values. The evaluations were conducted using a 5-point likert scale, with 5 representing the highest score.

Overall	GPT-4o	Reader 1	Reader 2	*P*-Value	*P*-Value	*P*-Value
				GPT-4o vs. R1	GPT-4o vs. R2	R1 vs. R2
Total	4 [2–5]	4 [1–5]	4 [1–5]	< 0.01	0.06	0.06
Center 1	4 [2–5]	4 [1–5]	4 [2–5]	0.1	0.1	0.8
Center 2	4.5 [2–5]	3 [1–5]	5 [1–5]	< 0.01	0.27	0.03
**Pancreas**						
Total	5 [2–5]	4 [1–5]	5 [2–5]	0.14	0.34	0.09
Center 1	5 [2–5]	4 [1–5]	5 [2–5]	0.11	0.10	0.11
Center 2	4.5 [2–5]	3.5 [2–5]	5 [2–5]	0.32	0.34	0.13
**Liver**						
Total	5 [2–5]	4 [2–5]	5 [2–5]	0.09	0.24	0.24
Center 1	4 [2–5]	4 [2–5]	4 [2–5]	1	0.86	0.86
Center 2	5 [2–5]	2 [2–5]	5 [2–5]	0.03	0.35	0.06
**Lung**						
Total	5 [2–5]	4 [2–5]	5 [2–5]	0.11	0.27	0.78
Center 1	4 [2–5]	4 [2–5]	4 [2–5]	0.20	0.60	0.60
Center 2	5 [4–5]	5 [2–5]	4.5 [2–5]	0.57	0.57	0.57
**Head/Neck**						
Total	4 [2–5]	4 [1–5]	3 [1–5]	0.16	0.17	0.78
Center 1	4 [3–5]	4 [2–5]	3 [2–5]	0.24	0.11	0.24
Center 2	4 [2–4]	3 [1–4]	3.5 [1–5]	0.504	0.91	0.65

Reader 1 = Resident with five years of experience. Reader 2 = board-certified radiologist with eight years of experience.

**Table 3:** Effect of reader, examination region, and center on the quality of follow-up recommendations, summarized by non-parametric relative treatment effects (RTE). Values are RTEs (0–1); 0.50 denotes no stochastic dominance versus the pooled distribution, values > 0.50 indicate higher (better) quality, and values < 0.50 indicate lower quality. P values refer to omnibus non-parametric tests comparing levels within each factor (reader, examination region, center); α = 0.05.

Effect of reader on quality (RTE)				
GPT-4o	Reader 1	Reader 2		P-value
0.56	0.43	0.51		< 0.01
**Effect of Examination on Quality (RTE)**				
Pancreas	Liver	Lung	Head/Neck	P-value
0.59	0.49	0.54	0.39	< 0.01
**Effect of Center on Quality (RTE)**				
Center 1	Center 2			P-value
0.50	0.50			0.91

*Reader 1 = Resident with five years of experience. Reader 2 = board-certified radiologist with eight years of experience.*

**Fig. 2:**
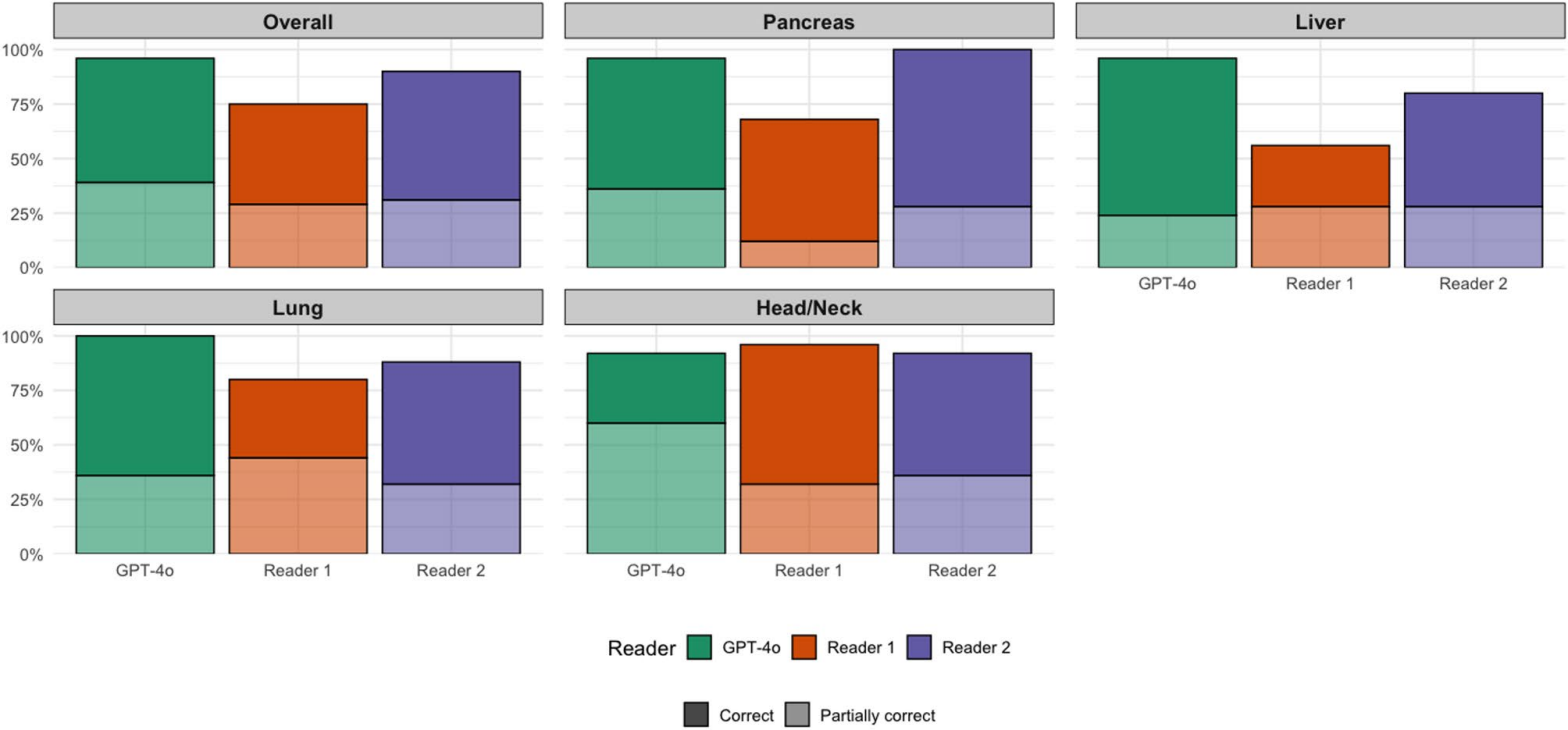
Overview of proposed follow-up examinations with correct (IIIa) or partially correct (IIIb) follow-up intervals as well as combined values (combined categories IIIa and IIIb), as determined by the expert consensus for the different subspecialities. Reader 1 = Resident with five years of experience. Reader 2 = board-certified radiologist with eight years of experience.

For correctness or partial correctness of the recommended follow-up interval (IIIa/IIIb combined), GPT-4o was accurate in 96% (96/100) of cases, compared with 75% (75/100) for R1 and 90% (90/100) for R2; two-proportion tests indicated significantly better performance for GPT-4o versus R1 (*p* < 0.001), and for R1 versus R2 (*p* = 0.005). There was no significant difference between GPT-4o and R2 (*p* = 0.096). GPT-4o showed the lowest overall rate of potentially harmful recommendations with 4% (4/100), compared with 25% for R1 (25/100) and 10% for R2 (10/100). Differences were most pronounced in liver and pancreatic imaging, where R1 exhibited substantially higher rates of potentially harmful recommendations, whereas GPT-4o and R2 demonstrated consistently low rates across subspecialties. Complete results can be found in Table 4 and are visualized in Fig. 2.

**Table 4:** Frequency of proposed follow-up examinations with correct (IIIa) or partially correct (IIIb) follow-up intervals as well as combined values (combined categories IIIa and IIIb), as determined by the expert consensus.

Overall	GPT4o			Reader 1			Reader 2			Σ
	Correct	Part. correct	Combined	Correct	Part. correct	Combined	Correct	Part. correct	Combined	
Total	57 (57%)	39 (39%)	96 (96%)	46 (46%)	29 (29%)	75 (75%)	59 (59%)	31 (31%)	90 (90%)	100
Center 1	35 (58,3%)	23 (38, 3%)	58 (96,7%)	27 (45%)	23 (38,3%)	50 (83,3%)	30 (50%)	26 (43,3%)	56 (93,3%)	60
Center 2	22 (55%)	16 (40%)	38 (95%)	19 (47,5%)	6 (15%)	25 (62,5%)	29 (72,5%)	5 (12,5%)	34 (85%)	40
**Pancreas**										
Total	15 (60%)	9 (36%)	24 (96%)	14 (56%)	3 (12%)	17 (68%)	18 (72%)	7 (28%)	25 (100%)	25
Center 1	9 (60%)	6 (40%)	15 (100%)	9 (60%)	3 (20%)	12 (80%)	9 (60%)	6 (40%)	15 (100%)	15
Center 2	6 (60%)	3 (30%)	9 (90%)	5 (50%)	0 (0%)	5 (50%)	9 (90%)	1 (10%)	10 (100%)	10
**Liver**										
Total	18 (72%)	6 (24%)	24 (96%)	7 (28%)	7 (28%)	14 (56%)	13 (52%)	7 (28%)	20 (80%)	25
Center 1	9 (60%)	5 (33, 3%)	14 (93,3%)	5 (33,3%)	7 (46,7%)	12 (80%)	6 (40%)	7 (46,7%)	13 (86,7%)	15
Center 2	9 (90%)	1 (10%)	10 (100%)	2 (20%)	0 (0%)	2 (20%)	7 (70%)	0 (0%)	7 (70%)	10
**Lung**										
Total	16 (64%)	9 (36%)	25 (100%)	9 36%)	11 (44%)	20 (80%)	14 (56%)	8 (32%)	22 (88%)	25
Center 1	9 (60%)	6 (40%)	15 (100%)	3 (20%)	9 (60%)	12 (80%)	9 (60%)	6 (40%)	15 (100%)	15
Center 2	7 (70%)	3 (30%)	10 (100%)	6 (60%)	2 (20%)	8 (80%)	5 (50%)	2 (20%)	7 (70%)	10
**Head/Neck**										
Total	8 (32%)	15 (60%)	23 (92%)	16 (64%)	8 (32%)	24 (96%)	14 (56%)	9 (36%)	23 (92%)	25
Center 1	8 (53,3%)	6 (40%)	14 (93,3%)	10 (66,7%)	4 (26,7%)	14 (93,3%)	6 (40%)	7 (46, 7%)	13 (86,7%)	15
Center 2	0 (0%)	9 (90%)	9 (90%)	6 (60%)	4 (40%)	10 (100%)	8 (80%)	2 (20%)	10 (100%)	10

Reader 1 = Resident with five years of experience. Reader 2 = board-certified radiologist with eight years of experience.

GPT-4o addressed all pathologies requiring follow-up in 92% (92/100), similar to R1 (91/100, 91%) and significantly higher than R2 (80/100, 80%); *p* = 0.014 for GPT4o vs. R1 and *p* = 0.027 for R1 vs. R2. The largest subspecialty gap occurred in liver examinations, where GPT-4o and R1 each achieved 22/25 (88%) and outperformed R2 with 16/25 (64%) (*p* = 0.047, respectively). There were no significant differences across the other subspecialties, while head/neck showed the highest completeness for GPT-4o at 24/25 (96%). For Category II (appropriateness of imaging modality), GPT-4o achieved 90% (90/100), which is slightly lower compared with 94% (94/100) for R1 and 95% (95/100) for R2, with no statistically significant differences observed overall (*p* = 0.297 for GPT-4o vs. R1; *p* = 0.197 for GPT-4o vs. R2; *p* = 0.756 for R1 vs. R2) or across subspecialties (Table 5).

**Table 5:** Absolute counts and percentages summarizing (i) whether all pathologies requiring radiologic follow-up were addressed (Category I: completeness of follow-up recommendation) and (ii) whether the selected imaging modality was appropriate (Category II: appropriateness of imaging modality), as determined by expert consensus.

Overall	All pathologies identified			Appropriate Modality			
	GPT-4o	Reader 1	Reader 2	GPT-4o	Reader 1	Reader 2	Σ
Total	92 (92%)	91 (91%)	80 (80%)	90 (90%)	94 (94%)	95 (95%)	100
Center 1	55 (91,7%)	56 (93,3%)	47 (78,3%)	53 (88,3%)	57 (95%)	58 (96,7%)	60
Center 2	37 (92,5%)	35 (87, 5%)	33 (82,5%)	37 (92,5%)	37 (92,5%)	37 (92,5%)	40
**Pancreas**							
Total	23 (92%)	23 (92%)	22 (88%)	23 (92%)	25 (100%)	25 (100%)	25
Center 1	13 (86,7%)	13 (86,7%)	13 (86,7%)	15 (100%)	15 (100%)	15 (100%)	15
Center 2	10 (100%)	10 (100%)	9 (90%)	8 (80%)	10 (100%)	10 (100%)	10
**Liver**							
Total	22 (88%)	22 (88%)	16 (64%)	21 (84%)	24 (96%)	23 (92%)	25
Center 1	14 (93,3%)	15 (100%)	10 (66,7%)	11 (73,3%)	14 (93,3%)	13 (86,7%)	15
Center 2	8 (80%)	7 (70%)	6 (60%)	10 (100%)	10 (100%)	10 (100%)	10
**Lung**							
Total	23 (92%)	23 (92%)	20 (80%)	24 (96%)	23 (92%)	25 (100%)	25
Center 1	13 (86,7%)	13 (86,7%)	10 (66,7%)	14 (93,3%)	13 (86,7%)	15 (100%)	15
Center 2	10 (100%)	10 (100%)	10 (100%)	10 (100%)	10 (100%)	10 (100%)	10
**Head/Neck**							
Total	24 (96%)	23 (92%)	22 (88%)	22 (88%)	22 (88%)	22 (88%)	25
Center 1	15 (100%)	15 (100%)	14 (93,3%)	13 (86,7%)	15 (100%)	15 (100%)	15
Center 2	9 (90%)	8 (80%)	8 (80%)	9 (90%)	7 (70%)	7 (70%)	10

Reader 1 = Resident with five years of experience. Reader 2 = board-certified radiologist with eight years of experience.

### Evaluation of the follow-up recommendations for different subspecialties

**Fig. 3:**
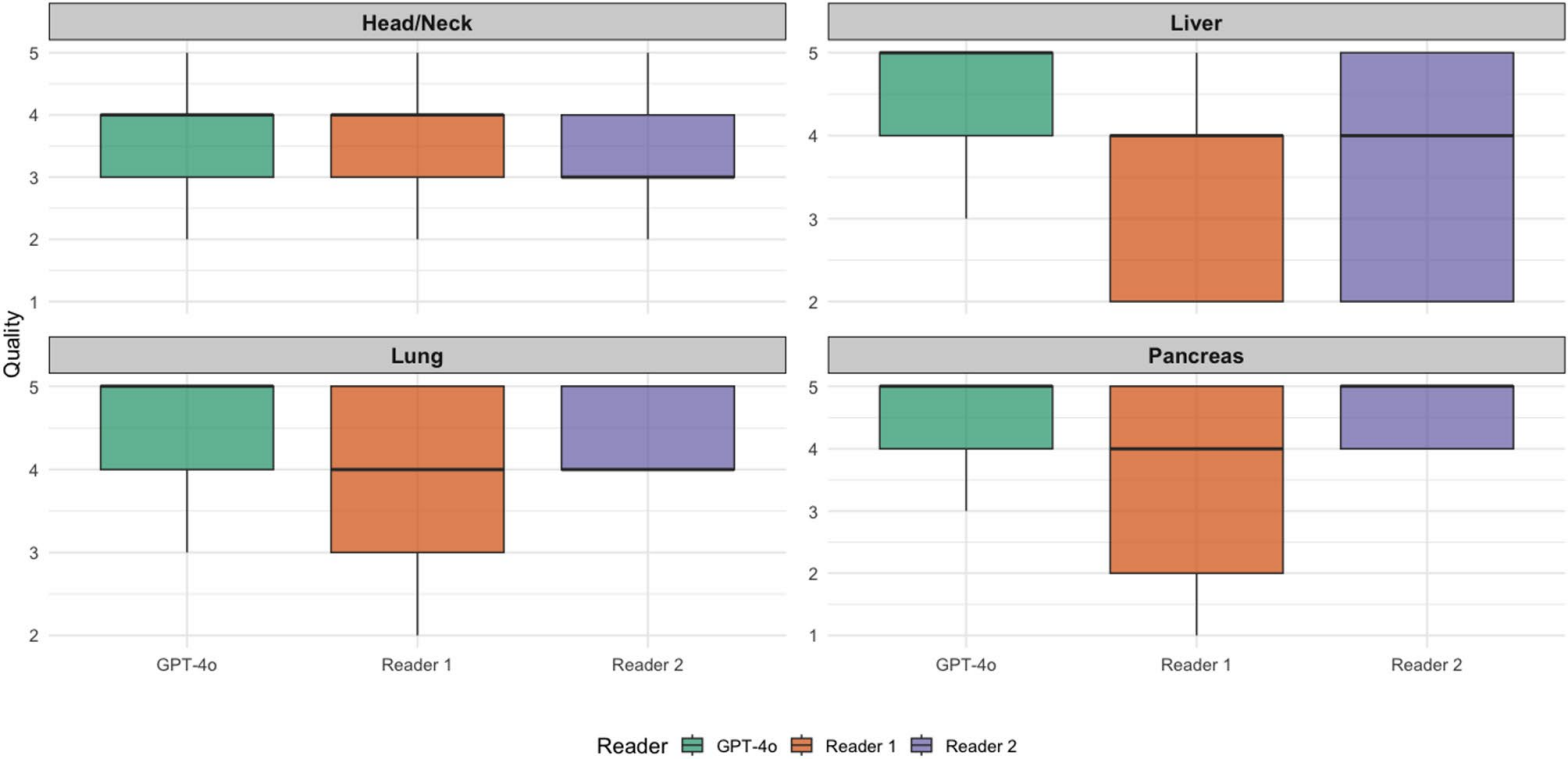
Visualization of overall follow-up quality for the different subspecialities. Reader 1 = Resident with five years of experience. Reader 2 = board-certified radiologist with eight years of experience.

A significant examination-region effect on quality was observed by RTE (pancreas :0.59; lung: 0.54; liver: 0.49; head/neck: 0.39; *p* < 0.01) (Table 2). A visualization of overall follow-up quality for the different subspecialities can be found in Fig. 3.

#### Pancreas

Quality medians were 5 (2–5) for GPT-4o, 4 (1–5) for R1, and 5 (2–5) for R2; pairwise comparisons showed no significant differences for GPT-4o versus R1 (*p* = 0.14) or GPT-4o versus R2 (*p* = 0.34). Correctness was 96% (24/25) for GPT-4o, 68% (17/25) for R1, and 100% (25/25) for R2; GPT-4o exceeded R1 (*p* = 0.010) without a significant difference versus R2 (*p* = 0.312), while R2 outperformed R1 (*p* = 0.002). Results showed slightly lower modality appropriateness for GPT-4o (23/25, 92%) vs. 25/25 (100%) for both readers (*p* = 0.149, respectively).

#### Liver

Quality medians were 5 (2–5) for GPT-4o, 4 (2–5) for R1, and 5 (2–5) for R2; pairwise comparisons were not significant for GPT-4o versus R1 (*p* = 0.09) or GPT-4o versus R2 (*p* = 0.24). Correctness was 96% (24/25) for GPT-4o, 56% (14/25) for R1, and 80% (20/25) for R2; GPT-4o was higher than R1 (*p* < 0.001) and not different from R2 (*p* = 0.082), and R1 did not differ from R2 (*p* = 0.069). GPT-4o yielded modality appropriateness for follow-up recommendation in 21/25 cases (84%), which was outperformed by R1 with 24/25 (96%) and by R2 with 23/25 (92%); *p* = 0.157 for GPT-4o vs. R1; *p* = 0.384 for GPT-4o vs. R2; *p* = 0.552 for R1 vs. R2.

#### Lung

Quality medians were 5 (2–5) for GPT-4o, 4 (2–5) for R1, and 5 (2–5) for R2; pairwise differences were not significant for GPT-4o versus R1 (*p* = 0.11) or GPT-4o versus R2 (*p* = 0.27). Correctness was 100% (25/25) for GPT-4o, 80% (20/25) for R1, and 88% (22/25) for R2; GPT-4o outperformed R1 (*p* = 0.018) and did not differ from R2 (*p* = 0.074), with no difference between R1 and R2 (*p* = 0.44).

#### Head/neck

Quality medians were 4 (2–5) for GPT-4o, 4 (1–5) for R1, and 3 (1–5) for R2; no significant differences were observed for GPT-4o versus R1 (*p* = 0.16) or GPT-4o versus R2 (*p* = 0.17). Correctness was 92% (23/25) for GPT-4o, 96% (24/25) for R1, and 92% (23/25) for R2; none of the pairwise comparisons reached significance (GPT-4o vs. R1, *p* = 0.55; GPT-4o vs. R2, *p* = 1.00; R1 vs. R2, *p* = 0.55).

### Evaluation of the follow-up recommendations by center

Comparing sites, there was no difference in overall quality of follow-up recommendations. The RTE was 0.50 for both center 1 and center 2 (*p* = 0.91), indicating identical distributions with no stochastic dominance of either center.

**Fig. 4:**
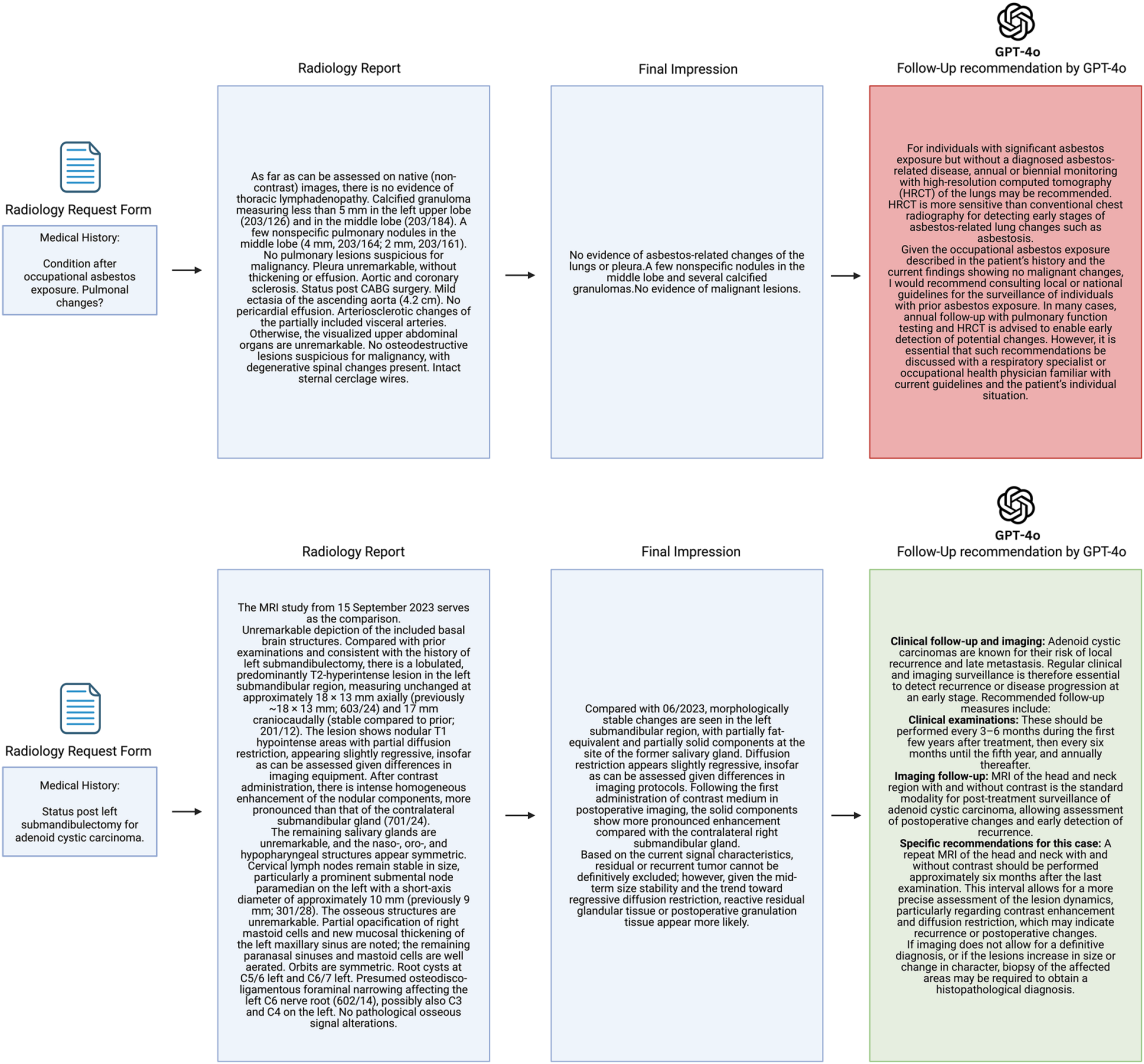
Exemplary cases of GPT-4o generated follow-up recommendation. In the first case (highlighted in red) the aortic ectasia was omitted, and the parenchymal lung framework changes were not addressed in detail: Expert Rating: 2 – Relevant Errors/potential harm. In the second case (highlighted in green), all follow-up recommendations were accurately reported according to the respective guidelines, including appropriate explanations related to the tumour entity: Expert Rating: 5 – No relevant errors/omissions.

Figure 4 presents representative examples of GPT-4o–proposed follow-up recommendations.

## Discussion

This retrospective study investigated the potential of GPT-4o, a generic LLM, for accurate determination of follow-up examination modality and timeframe for radiology reports of different subspecialties from clinical routine. Results were compared to the performance of a radiology resident and a more experienced board-certified radiologist. Follow up recommendations were assessed by two board-certified radiologists based on the recommendations from internationally recognized radiological and cancer societies. GPT-4o generated follow-ups with overall quality comparable to an experienced radiologist and superior to a trainee. The model excels with high completeness and generally appropriate follow up timing and modality with RTE showing a positive, significant reader effect for GPT-4o. Regarding subspecialties, the LLM excels at pancreas and lung imaging, whereas performance for head & neck examination was lower for all readers. These findings highlight the potential of GPT-4o to function as decision support for standardized, guideline-aligned follow-up.

Current evidence on LLM-driven follow-up recommendations in radiology is early but encouraging across distinct use cases. In chest CT reporting, a large single-center study (1009 reports) found GPT-4o-mini and ERNIE-4.0-Turbo-8 K generated guideline-concordant pulmonary-nodule follow-up in 92.8% and 94.6% of cases, respectively (*p* = 0.07), with harmful recommendations ≤ 3.5%, yet the authors emphasized the need for human oversight and rigorous validation^21^. Further, for incidental pancreatic cysts, a multimodal GPT-4o workflow that embedded flowcharts and tables from the ACR Incidental Findings Committee algorithm markedly improved recommendation accuracy (90%–92%) versus default knowledge (40%–42%) or plain-text retrieval-augmented generation (24%–25%), as rated by three radiologists. In a second task the LLM correctly adjusted schedules in 81% of cases when prior reports were provided, illustrating that structured knowledge ingestion and vision-to-text can be pivotal for complex guideline adherence^22^. Finally, in a perioperative setting outside diagnostic radiology, a small prospective evaluation in oral and maxillofacial surgery showed GPT-4 achieved top scores for correctness and reasonableness across 30 common follow-up questions^23^. Building on those previous studies, our work incorporates data from two centres to strengthen the robustness of the findings. Furthermore, to the best of our knowledge, no prior research has systematically compared translation performance across different imaging subspecialities. In each subspecialty, readers were provided only the complete radiology reports, without appended guideline recommendation. This more accurately reflects real-world clinical practice, where identification of the relevant pathology, guideline selection, and evidence retrieval are essential elements of radiologic decision-making.

Our findings suggest that GPT-4o can reliably identify reportable pathologies that warrant follow-up and map them to appropriate guideline frameworks to propose modality and interval. The model’s stronger performance in lung, pancreas, and liver likely reflects the high degree of standardization in these domains as the Fleischner criteria for pulmonary nodules^2^, LI-RADS for hepatocellular carcinoma^24^, and the Fukuoka consensus for IPMN^19^, where decision pathways are well codified and widely adopted. By contrast, head and neck imaging exhibited lower performance for all raters, plausibly owing to greater clinical heterogeneity and the coexistence of partially divergent recommendations across societies (e.g., NCCN, ACR, and regional oncology groups), as well as site-specific differences in sequence selection related to equipment availability and software constraints. Notably, performance did not differ by center, indicating that local reporting styles and institutional context had minimal impact on follow-up quality and supporting the potential generalizability of GPT-4o–assisted recommendations across practice settings.

Our findings provide evidence supporting further evaluation and potential implementation of GPT-4o as a clinical decision support tool in medical workflows. A variety of imaging examinations require follow-up imaging to monitor disease progress or effectiveness of a given therapy. This is especially the case for oncologic diseases. Eventually, the artificial intelligence could be locally integrated as a semi-automatic tool^25^, providing follow-up recommendations to attending radiologists that only need confirmation. In this step the radiologist remains essential, as GPT-4o may occasionally misinterpret reports and generate inappropriate and harmful follow up recommendations. With an interface to the examination schedule, the appointments could then be seamlessly integrated, eliminating the need for additional steps. This interface could be used to schedule imaging recommendations automatically. In the context of magnetic resonance imaging, this could aid to schedule examinations of the same body regions consecutively. This could streamline examination schedules and improve efficiency as it eliminates the need to change coils in between patients. Further, a tool promoting standardized follow-up recommendations could counteract potential inefficiencies related to the current variability in radiological practice^3,4^. Patients may undergo less unnecessary imaging (e.g. incurring avoidable cost and radiation exposure), while others might receive timelier follow-up when it is warranted.

Summarized, the LLM can serve as a valuable support system for determination of follow-up examination and scheduling. This would reduce patient waiting times, alleviate growing workloads, optimize the allocation of medical resources, and ultimately enhance patient care. Moreover, when incorporating GPT-4o into medical workflows as a medical software device, an application programming interface-based implementation should be prioritized to ensure more consistent and standardized prompting behaviour. While comparable or superior performance may be achievable with other or future, more medically tailored LLMs, generalizability is not guaranteed and likely depends on factors such as medical domain adaptation, guideline exposure, and robustness to prompt variation. Importantly, claims of transferability across models require prospective validation and safety-focused evaluation before clinical deployment. Notwithstanding, LLMs pose significant data privacy concerns when processing sensitive medical information on external cloud-based platforms. These risks must be addressed by solely deploying models on secure, locally hosted servers that enable strict compliance with data protection regulations.

This work has several limitations, many of which are inherent to current large language models. GPT-4o, like related systems, generates language without genuine semantic understanding, which can yield plausible but incorrect statements and occasional inconsistencies. Model opacity further constrains interpretability: the provenance of training data and the specific guideline sources implicitly encoded by the model are unknown, limiting auditability and reproducibility of decisions. Future research should prioritize architectures and deployment frameworks with explicit source transparency (e.g., guideline-linked retrieval traces) to enable verification and critical appraisal. Methodologically, the retrospective design may introduce selection and information biases. Even with a consensus reading by two experts and the relatively straightforward follow-up imaging recommendations by internationally recognized radiological societies, the choice of reference standard presents a limitation in the study design. Further, the cohort was relatively small (*n* = 100) and intentionally heterogeneous, covering four subspecialties and multiple guideline frameworks. While this design enabled a cross-domain proof-of-concept evaluation, it limited statistical power for subgroup analyses and generalizability beyond adult oncologic CT/MRI reporting. Consequently, smaller between-reader differences may not have been detectable, and near-threshold results (e.g. *p* = 0.06 for GPT4o vs. R2 in overall follow-up quality) may be attributable to limited power rather than the absence of an effect. Moreover, the balanced 25-per-subspecialty sampling does not reflect routine clinical prevalence and therefore limits extrapolation to other subspecialties, non-oncologic indications, emergency settings, or different languages and reporting styles. The scope of our study was focused on radiology reports only and therefore evaluations were conducted exclusively by radiologists. Thus, our findings do not reflect other kinds of medical records and perspectives of other medical professionals. Given the risk of misinterpreting radiology reports and producing inappropriate recommendations, the educational standard and final authority should continue to rest with board-certified radiologists, clinicians and established clinical guidelines.

In conclusion, GPT-4o produced follow-up recommendations with overall quality comparable to a board-certified radiologist and higher than a radiology resident, characterized by high completeness and generally appropriate modality and interval selection. Performance varied modestly by subspecialty, with higher accuracy in more standardized domains (liver, pancreas, lung) and comparatively lower performance in head and neck. These results support the use of GPT-4o as decision support to promote standardized, guideline-aligned follow-up. Future work (i) should assess whether an LLM can automatically flag radiology reports with insufficient information to determine follow-up, thereby streamlining workflow, and (ii) evaluate locally adapted deployments that integrate national and institutional guidelines. Prospective, international multicenter studies will be essential to validate these findings and to establish generalizability across diverse practice settings.

## Supplementary Information

Below is the link to the electronic supplementary material.

Supplementary Material 1

## Data Availability

The data that support the findings of this study are currently not openly available due to reasons of sensitivity and are available including the ratings from the corresponding author upon reasonable request.

## Abbreviations

AI: Artificial intelligence
ACR: American College of Radiology
ACS: American Cancer Society
AJNR: American Journal of Neuroradiology
GPT-4o: Generative pre-trained transformer 4omni
LLM: Large language model
NCCN: National comprehensive cancer network
RRF: Radiology request form
