# Supplementary material for: GPT-4o for Automated Determination of Follow-up Examinations Based on Radiology Reports from Clinical Routine
Source: Sci Rep. 2026 Apr 16;16:12587. doi: 10.1038/s41598-026-40317-9 (PMC13087124; doi:10.1038/s41598-026-40317-9)
Supplement: Supplementary file 1 — Supplementary Material 1 [file 41598_2026_40317_MOESM1_ESM.docx]

| **Examination** | **Center** | **Subject 1** | **Subject 2** | **N pairs** | **SD diff.** | **MDE (Cohen`s d_z_)** | **MDE (quality points)** | **alpha** | **power** |
| --- | --- | --- | --- | --- | --- | --- | --- | --- | --- |
| Head/Neck | Center 1 | GPT-4o | Reader 1 | 15 | 0.9759 | 0.778029 | 0.759279 | 0.05 | 0.8 |
| Head/Neck | Center 1 | GPT-4o | Reader 2 | 15 | 1.046536 | 0.778029 | 0.814236 | 0.05 | 0.8 |
| Head/Neck | Center 1 | Reader 1 | Reader 2 | 15 | 1.046536 | 0.778029 | 0.814236 | 0.05 | 0.8 |
| Head/Neck | Center 2 | GPT-4o | Reader 1 | 10 | 0.971825 | 0.996004 | 0.967942 | 0.05 | 0.8 |
| Head/Neck | Center 2 | GPT-4o | Reader 2 | 10 | 1.595131 | 0.996004 | 1.588758 | 0.05 | 0.8 |
| Head/Neck | Center 2 | Reader 1 | Reader 2 | 10 | 1.776388 | 0.996004 | 1.769291 | 0.05 | 0.8 |
| Liver | Center 1 | GPT-4o | Reader 1 | 15 | 1.812654 | 0.778029 | 1.410297 | 0.05 | 0.8 |
| Liver | Center 1 | GPT-4o | Reader 2 | 15 | 1.579632 | 0.778029 | 1.229 | 0.05 | 0.8 |
| Liver | Center 1 | Reader 1 | Reader 2 | 15 | 1.869556 | 0.778029 | 1.454568 | 0.05 | 0.8 |
| Liver | Center 2 | GPT-4o | Reader 1 | 10 | 1.449138 | 0.996004 | 1.443347 | 0.05 | 0.8 |
| Liver | Center 2 | GPT-4o | Reader 2 | 10 | 1.264911 | 0.996004 | 1.259857 | 0.05 | 0.8 |
| Liver | Center 2 | Reader 1 | Reader 2 | 10 | 1.581139 | 0.996004 | 1.574821 | 0.05 | 0.8 |
| Lung | Center 1 | GPT-4o | Reader 1 | 15 | 1.121224 | 0.778029 | 0.872345 | 0.05 | 0.8 |
| Lung | Center 1 | GPT-4o | Reader 2 | 15 | 1.486447 | 0.778029 | 1.156499 | 0.05 | 0.8 |
| Lung | Center 1 | Reader 1 | Reader 2 | 15 | 1.447494 | 0.778029 | 1.126192 | 0.05 | 0.8 |
| Lung | Center 2 | GPT-4o | Reader 1 | 10 | 1.354006 | 0.996004 | 1.348596 | 0.05 | 0.8 |
| Lung | Center 2 | GPT-4o | Reader 2 | 10 | 1.686548 | 0.996004 | 1.679809 | 0.05 | 0.8 |
| Lung | Center 2 | Reader 1 | Reader 2 | 10 | 1.159502 | 0.996004 | 1.154869 | 0.05 | 0.8 |
| Pancreas | Center 1 | GPT-4o | Reader 1 | 15 | 0.99043 | 0.778029 | 0.770584 | 0.05 | 0.8 |
| Pancreas | Center 1 | GPT-4o | Reader 2 | 15 | 0 | 0.778029 | 0 | 0.05 | 0.8 |
| Pancreas | Center 1 | Reader 1 | Reader 2 | 15 | 0.99043 | 0.778029 | 0.770584 | 0.05 | 0.8 |
| Pancreas | Center 2 | GPT-4o | Reader 1 | 10 | 2.213594 | 0.996004 | 2.20475 | 0.05 | 0.8 |
| Pancreas | Center 2 | GPT-4o | Reader 2 | 10 | 1.354006 | 0.996004 | 1.348596 | 0.05 | 0.8 |
| Pancreas | Center 2 | Reader 1 | Reader 2 | 10 | 2.097618 | 0.996004 | 2.089236 | 0.05 | 0.8 |

**Supplementary Table 1**. Minimum detectable effect by exam and center.

*Legend: MDE = minimum detectable effect; SD = standard deviation*

| **Overall** | **Subject 1** | **Subject 2** | **N pairs** | **SD diff.** | **MDE (Cohen`s d_z_)** | **MDE (quality points)** | **alpha** | **power** |
| --- | --- | --- | --- | --- | --- | --- | --- | --- |
| All | GPT-4o | Reader 1 | 100 | 1.450148 | 0.282901 | 0.410248 | 0.05 | 0.8 |
| All | GPT-4o | Reader 2 | 100 | 1.326345 | 0.282901 | 0.375224 | 0.05 | 0.8 |
| All | Reader 1 | Reader 2 | 100 | 1.580787 | 0.282901 | 0.447206 | 0.05 | 0.8 |

**Supplementary Table 2**. Minimum detectable effect overall.

*Legend: MDE = minimum detectable effect; SD = standard deviation*

| **Center** | **Subject 1** | **Subject 2** | **N pairs** | **SD diff.** | **MDE (Cohen`s d_z_)** | **MDE (quality points)** | **alpha** | **power** |
| --- | --- | --- | --- | --- | --- | --- | --- | --- |
| Center 1 | GPT-4o | Reader 1 | 60 | 1.259876 | 0.367708 | 0.463267 | 0.05 | 0.8 |
| Center 1 | GPT-4o | Reader 2 | 60 | 1.197455 | 0.367708 | 0.440314 | 0.05 | 0.8 |
| Center 1 | Reader 1 | Reader 2 | 60 | 1.39521 | 0.367708 | 0.51303 | 0.05 | 0.8 |
| Center 2 | GPT-4o | Reader 1 | 40 | 1.647842 | 0.454273 | 0.748569 | 0.05 | 0.8 |
| Center 2 | GPT-4o | Reader 2 | 40 | 1.514883 | 0.454273 | 0.68817 | 0.05 | 0.8 |
| Center 2 | Reader 1 | Reader 2 | 40 | 1.77157 | 0.454273 | 0.804776 | 0.05 | 0.8 |

**Supplementary Table 3**. Minimum detectable effect by center.

*Legend: MDE = minimum detectable effect; SD = standard deviation*

| **Examination** | **Subject 1** | **Subject 2** | **N pairs** | **SD diff.** | **MDE (Cohen`s d_z_)** | **MDE (quality points)** | **alpha** | **power** |
| --- | --- | --- | --- | --- | --- | --- | --- | --- |
| Head/Neck | GPT-4o | Reader 1 | 25 | 0.957427 | 0.584027 | 0.559163 | 0.05 | 0.8 |
| Head/Neck | GPT-4o | Reader 2 | 25 | 1.293574 | 0.584027 | 0.755482 | 0.05 | 0.8 |
| Head/Neck | Reader 1 | Reader 2 | 25 | 1.398809 | 0.584027 | 0.816943 | 0.05 | 0.8 |
| Liver | GPT-4o | Reader 1 | 25 | 1.951068 | 0.584027 | 1.139477 | 0.05 | 0.8 |
| Liver | GPT-4o | Reader 2 | 25 | 1.443376 | 0.584027 | 0.842971 | 0.05 | 0.8 |
| Liver | Reader 1 | Reader 2 | 25 | 1.938212 | 0.584027 | 1.131969 | 0.05 | 0.8 |
| Lung | GPT-4o | Reader 1 | 25 | 1.193035 | 0.584027 | 0.696765 | 0.05 | 0.8 |
| Lung | GPT-4o | Reader 2 | 25 | 1.557776 | 0.584027 | 0.909784 | 0.05 | 0.8 |
| Lung | Reader 1 | Reader 2 | 25 | 1.351542 | 0.584027 | 0.789337 | 0.05 | 0.8 |
| Pancreas | GPT-4o | Reader 1 | 25 | 1.556706 | 0.584027 | 0.909159 | 0.05 | 0.8 |
| Pancreas | GPT-4o | Reader 2 | 25 | 0.866025 | 0.584027 | 0.505782 | 0.05 | 0.8 |
| Pancreas | Reader 1 | Reader 2 | 25 | 1.535144 | 0.584027 | 0.896566 | 0.05 | 0.8 |

**Supplementary Table 4**. Minimum detectable effect by examination.

*Legend: MDE = minimum detectable effect; SD = standard deviation*
